# Implications of Socio-Cultural Pressure for a Thin Body Image on Avoidance of Social Interaction and on Corrective, Compensatory or Compulsive Shopping Behaviour

**DOI:** 10.3390/ijerph20043567

**Published:** 2023-02-17

**Authors:** António Azevedo, Ângela Sá Azevedo

**Affiliations:** 1 Lab2PT, Landscape, Heritage and Territory Laboratory Research Unit, School of Economics and Management, University of Minho, 4710-057 Braga, Portugal; 2 Faculty of Philosophy and Social Sciences, Centre for Philosophical and Humanistic Studies, Universidade Católica Portuguesa, 4710-297 Braga, Portugal

**Keywords:** body image, sociocultural attitudes towards appearance, acceptance of cosmetic surgery scale, compulsive buying behaviour, social-interaction avoidance, compensatory consumption

## Abstract

This paper aims to discuss the implications of body talk and socio-cultural pressure for the internalisation of a thin body image in purchase decisions, shopping habits and other outcomes of body dissatisfaction, in particular the proneness to avoid human/social interaction in retail contexts and proneness to engage in corrective, compensatory or compulsive shopping behaviour. This paper conducted an online questionnaire that measured the following constructs: body mass index; Socio-cultural Attitudes Towards Appearance Scale-4 (SATAQ-4), Body Appreciation Scale (BAS-2), Acceptance of Cosmetic Surgery Scale (ACSS), Compulsive Buying Follow-up Scale (CBFS), proneness to avoid social interaction in retail contexts, and the intention to purchase a list of products and services as a compensation for body dissatisfaction. A structural equations model supported the hypotheses proposing the influence of BAS-2 and SATAQ-4 (the internalisation of thin/athletic body and the social comparison induced by family, peers and media) upon the outcomes of social-interaction avoidance, ACSS and CBFS. Nevertheless, BAS-2 only influences social-interaction avoidance. This paper provides several recommendations to brand managers highlighting the social responsibility role of brand advertising in enhancing positive body appreciation, mitigating the psychological damage caused by socio-cultural pressure and preventing the stigmatisation bias against obese people.

## 1. Introduction

According to the systematic review of Allen and Robson [1], body dissatisfaction is common among men and women in developed and developing nations [2] with prevalence estimates ranging from 11 to 72% among women and 8–61% among men [3]. Some studies estimate that 25% to 80% of people are dissatisfied with their physical appearance, wherein the population of young people and adolescents is at greatest risk for the development of disorders [4,5]. Obesity and being overweight are often the principal reasons for body dissatisfaction [6,7,8,9,10].

Furthermore, positive body image is a broad concept that includes love and respect for the body and allows individuals to appreciate its uniqueness and functionality [11]. People with a positive body image are more likely to accept their perceived body imperfections or deviations from cultural ideals, have a mindful connection with their body’s needs and interpret incoming information in a body-protective manner. Moreover, Tylka and Wood-Barcalow [12] defined body appreciation as “accepting, holding favourable opinions toward and respecting the body, while also rejecting media-promoted appearance ideals as the only form of human beauty”.

This paper aims to discuss the implications of body talk and socio-cultural pressure for positive body image on buying decision-making processes, on consumer behaviour preferences and shopping styles and on the potential consequences of body dissatisfaction, in particular the proneness to avoid human/social interaction in retail contexts and the proneness to change the shopping basket and engage in corrective/compensatory/compulsive shopping behaviour.

From a marketing perspective, research has tended to focus on issues such as how advertising and promotions influence consumers’ food consumption [13,14] and the impacts of social marketing interventions on food consumption practices and obesity prevention campaigns [15,16,17] and exercise behaviour [18]. Furthermore, social marketing researchers also have dedicated some attention to body image [19] and the role of body beauty in advertising endorsement [20].

This paper proposes an innovative research topic that makes a bridge between two scientific realms and their respective bodies of literature—(clinical) psychology related to obesity and eating disorders and fields of knowledge related to marketing and consumer behaviour. This intersection is a research gap that still requires attention from researchers [21].

The paper is organized as follows: based on the literature review, the next section proposes a new conceptual model that formulates several research hypotheses about the relationships between the antecedents and consequences of body appreciation/dissatisfaction. The third chapter describes the methodology of a quantitative study that aims to validate the model whose implications are discussed in the fourth chapter. The last chapter provides new insights and recommendations to brand and retail managers about the need to adapt the marketing mix in order to satisfy the needs and concerns of two target groups of consumers: (a) consumers who have positive body image but are still exposed to the socio-cultural pressure from their family, peers and media, which contributes to the internalisation of a thin, lean or muscular body as a social norm; (b) consumers who feel uncomfortable with their bodies or have low levels of self-rated body appreciation and alter their shopping behaviour habits and preferences as a consequence of that body dissatisfaction.

## 2. Literature Review

### 2.1. Positive Body Image Versus Body Image Dissatisfaction

Positive body image is a complex concept, defined by Wood-Barcalow et al. [22]:

*“An overarching love and respect for the body that allows individuals to (a) appreciate the unique beauty of their body and the functions that it performs for them; (b) accept and even admire their body, including those aspects that are inconsistent with idealised images; (c) feel beautiful, comfortable, confident and happy with their body, which is often reflected as an outer radiance, or a “glow;” (d) emphasise their body’s assets rather than dwell on their imperfections; and (f) interpret incoming information in a body-protective manner whereby most positive information is internalised and most negative information is rejected or reframed”.*

According to Tylka and Wood-Barcalow [11], a positive body image is a distinct construct from a negative body image and comprises the following aspects: (a) it is multifaceted (including body appreciation, body acceptance and love, adaptive appearance investment, broadly conceptualising beauty, inner positivity that radiates outward and manifests as adaptive behaviour and filtering information in a body-protective manner); (b) it is holistic (in which internal experiences, such as inner positivity and protective filtering, are interwoven with external behaviour, interpersonal relationships, community, media and culture to create attunement); (c) it is stable but adjustable via intervention, likely protective, linked to unconditional body acceptance by others and moulded by individuals’ multiple social identities.

Tylka and Wood-Barcalow [11] also elucidate that positive body image is not: (a) being highly satisfied with all aspects of appearance; (b) limited to appearance to the exclusion of other body dimensions (e.g., body functionality); (c) expressed as narcissism or vanity; (d) foolproof in its ability to protect against all body-image-related threats, linked to disengagement from selfcare, or aided by frequent appearance-related compliments from others.

Physiological, biological, psychological and sociological aspects are involved in structuring this concept [4,23]. The influence of social relations and the media in the formation of body image has been strongly associated with body dissatisfaction. Body image concerns may be impacted by several factors, such as body mass index (BMI), pubertal status and external influence from parents, peers and the media through appearance comparison and internalisation of the thin ideal [24].

Several self-report questionnaires have been developed to assess body dissatisfaction, such as the Body Investment Scale of Orbach and Mikulincer [25], the Body Image Questionnaire, refined by Penelo et al. [26], the Body Image Avoidance Questionnaire of Rosen et al. [27] and the Male Body Attitudes Scale (MBAS), developed by Tylka, Bergeron and Schwartz [28]. On the other hand, the most widely-used measure of body appreciation is the Body Appreciation Scale-2 (BAS-2), created by Avalos, Tylka and Wood-Barcalow [29].

In general, women experience greater body dissatisfaction than men [30], while men face unique muscularity-focused body concerns. The male body ideal includes two dimensions: leanness and muscularity [31,32,33,34]. Allen and Robson [1] also investigated the influence of personality traits and found that higher levels of neuroticism and lower levels of extraversion and conscientiousness are associated with a greater risk of body dissatisfaction in men and women, regardless of their actual body weight.

Body dissatisfaction may be related to the development of eating disorders and body dysmorphic disorder [35], unhealthy practices for weight control [36,37,38], impaired sexual function [39], low self-esteem [40], interpersonal difficulties, depressed mood, social anxiety [41], low physical activity, substantial morbidity, stress, substance abuse and obesity [42,43]. Since 2000, the DSM-IV-TR (APA, 2000) includes over-concern with weight and shape as a criterion for the diagnosis of anorexia nervosa and bulimia nervosa [44,45].

### 2.2. Body Talk and Socio-Cultural Attitude towards Physical Appearance: Internalisation of Thin/Athletic Bodies, Family, Peers and Media/Advertising Pressures

Body talk is defined as the way in which individuals engage in mutual disclosure of thoughts, emotions or attitudes about their own body with a conversation partner; this talk may include negative talk (e.g., “My thighs are too fat”) and positive talk (e.g., “I like how my body looks”). Exposure to negative body talk is a key driver of negative body image [46] resulting from friends’ social network posting [47,48,49] or the fear of fat [9]. Body talk is moderated by gender [34,49] or ethnic–racial identity [50].

The most frequently used model to explain the influence of socio-cultural pressures on body dissatisfaction is the tripartite model proposed by Thompson et al. [51]. This model suggests that individuals are pressured to achieve ideals of culturally adopted appearance primarily due to three social influences (family, friends and media). Internalisation refers to the extent to which an individual accepts the ideals of appearance and expresses the desire to achieve them, starting to have thoughts and behaviour that aim to meet this ideal which, in most cases, is unattainable [4].

In order to measure socio-cultural influences on body dissatisfaction, Schaefer et al. [38,52] developed the Socio-cultural Attitudes Towards Appearance Scale-4 (SATAQ-4) based on 22 items comprising five dimensions: internalisation of thin/low body fat, internalisation of muscular/athletic body, family pressure, peer pressure and media pressure.

Family is one of the most important socialisation agents predicting body dissatisfaction and eating disorder symptoms and family communication is a major mechanism of parental influence on the body-image disturbances of adolescent daughters [8,53].

Moreover, the influence of peers and friends has been confirmed by several studies [9]. For example, Arroyo and Brunner [47] verified that friends’ fitness posts on social networks (SNS) were positively associated with negative body talk and that this relationship was strongest for individuals who reported a higher tendency to compare themselves to others, even after controlling for body satisfaction, healthy eating, exercise behaviour and frequency of SNS use.

Huang, Peng and Ahn [54] conducted a meta-analysis in which they examined the relationship between media pressure and a wide range of body-image-related outcomes as well as moderators (e.g., media type, outcome type, age group, gender proportion in the sample and study design). These authors found that both women and men of all age groups across multiple countries could be affected by thin/athletic-ideal media exposure [7]. Moreover, media with a commercial purpose compared to those with no commercial purpose have been less effective in increasing body image concerns and media exposure has been the most influential factor in provoking eating disorders and the internalisation of the thin ideal.

According to Brown and Tiggemann [55], exposure to images of thin fashion models contributes to women’s body dissatisfaction. These authors have investigated the impact of attractive celebrities and peer images on women’s body image. Social comparison theory [56] argues that humans have an innate drive to compare themselves with others in order to make evaluations about their abilities and opinions, especially when objective measures do not exist. When comparing their appearance with that of thin and attractive models, most women will inevitably fall short, resulting in negative feelings about themselves and their bodies. Experimental research has confirmed that the observed negative effect of media exposure on negative mood and body dissatisfaction is at least partly mediated by social comparison processing [57,58,59]. In addition, celebrity worship moderates an increased effect of celebrity images on body dissatisfaction [55]. Slater, Cole and Fardouly [60] have demonstrated that acute exposure to parody images led to increased body satisfaction and positive mood (happiness) compared to exposure solely to thin-ideal celebrity images. The findings provide preliminary support for the use of humorous, parody images for improving body satisfaction and a positive mood in young women.

The same negative effect occurs with men exposed to advertising, who are increasingly troubled with weight loss, with a strong focus on the development of lean, muscular and generally athletic physiques [37,61]. The internalisation of societal standards of attractiveness is known to play a role in the development of body dissatisfaction and eating disorders.

**Figure 1:**
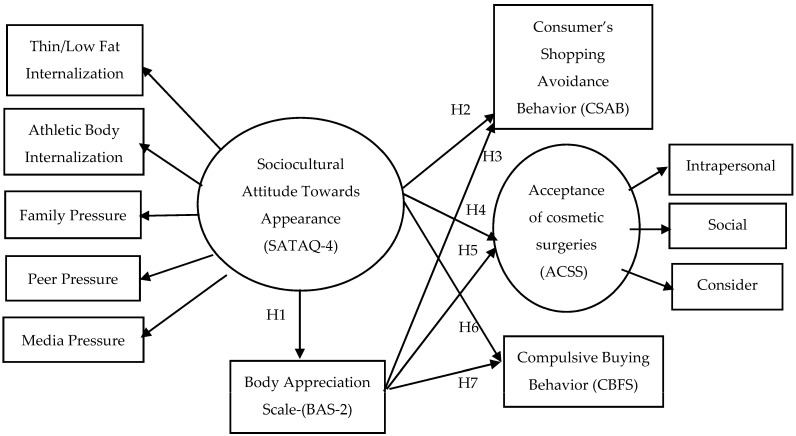
Conceptual model of the assessment of shopping behaviour implications resulting from body dissatisfaction (study no. 1).

Swami et al. [62], Jovic et al. [63] and Sundgot-Borgen et al. [64], among others, found a negative correlation between socio-cultural pressure (SATAQ) dimensions and body appreciation. Therefore, this paper proposes the first research hypothesis, represented in the conceptual model in Figure 1.

**(H1).**  *Socio-cultural Attitudes towards Appearance (measured by SATAQ-4), as a composite of five dimensions (internalisation of thin/low body fat, internalisation of muscular/athletic body, family pressure, peer pressure and media pressure) is negatively correlated with the self-perception of body image (measured with BAS-2).*

According to Vartanian and Hopkinson [65], conformity appears to be a risk factor for internalisation of societal standards of attractiveness and could be targeted in efforts to reduce internalisation, negative body image and disordered eating. Conformity was defined as an “involving characteristic willingness to identify others and emulate them, to give into others to avoid conflict and generally, to be a follower rather than a leader in terms of ideas, values and behaviours” [66]. On the other hand, Tompkins et al. [67] experimentally examined how conformity to fat talk, or its seemingly opposite form of dialogue—positive body talk—increases social likeability in female conversations.

### 2.3. Implications of Socio-Cultural Pressure for Thin Body Image in Consumers’ Information Processing and Purchase Decisions

#### 2.3.1. Social Visibility’s Avoidance and Proneness for E-Commerce and Non-Conspicuous Consumption

This paper also aims to discuss the consequences and implications of socio-cultural pressure for positive body image in consumers’ information processing and purchase decisions. People who have a self-ascribed negative image are often concerned about what others think about them, because they are often facing widespread discrimination [68,69].

The first implication is obvious—if an individual has a negative body image he/she will avoid the social visibility associated with shopping in high-street shops or in shopping centres, thereby avoiding all kinds of social exposure inherent to human/social interaction with the shop staff and other shoppers [70,71].

On other hand, conspicuous consumption is a “deliberate engagement in symbolic and visible purchase, possession and usage of products and services imbued with scarce economic and cultural capital with the motivation to communicate a distinctive self-image to others” [72]. According to Bronner and de Hoog [73], there are three recent trends in consumer behaviour that have increased the proneness for conspicuous and experiential consumption. Although this type of consumption is more tied to self-development, social visibility continues to be the main driver. This paper therefore assumes that consumers with a negative body image will want to avoid social visibility and consequently the social benefits of conspicuous consumption. Moreover, according to Barauskaite et al. [74], conspicuous consumption and self-control motivation may positively influence engagement with healthy nutrition habits solely in order impress peers and friends.

On the other hand, according to Neave, Tzemou and Fastoso [75], for grandiose narcissists, conspicuous consumption will be driven by their need for uniqueness, whilst that of vulnerable narcissists by their need to avoid social disapproval. Grandiose narcissists are extraverted, exhibitionistic, self-assured, aggressive and dominant, whereas vulnerable narcissists show high levels of introversion, anxiety and defensiveness [76].

Therefore, the conceptual model proposed in this paper (Figure 1) suggests two hypotheses, translating the negative correlation between proneness to avoid social exposure in an offline retail context as a consequence of socio-cultural pressure and body appreciation:

**(H2).**  *Socio-cultural Attitudes Towards Appearance (SATAQ-4) is positively correlated with consumers’ intention to avoid social interaction with retail salespersons and other consumers in a retail environment (high-street shops or shopping centres).*

**(H3).**  *Body image appreciation (BAS-2) is negatively correlated with consumers’ intention to avoid social interaction with retail salespersons and other consumers in a retail environment (high-street shops or shopping centres).*

Consequently, consumers with a negative body image will prefer to shop in the e-commerce channel and benefit from all the advantages resulting from the higher privacy and lower social visibility of the online environment. Moreover, augmented reality (AR)-based virtual try-on product presentations allow consumers to assess how well the displayed products match their actual bodies, unlike traditional web-based product presentations. Yim and Park [77] reveal that consumers who perceive their body image as unfavourable evaluate AR presentations more favourably than traditional web-based product presentations, while consumers who perceive their body image as favourable record no differences in their responses to the two presentations.

#### 2.3.2. Changes in Products and Services Shopping Basket and “Corrective” Buying Preferences

Individuals with a negative body image may also change their shopping habits in several product categories, besides obvious products/services such as the acquisition of weight-loss products, having good eating habits [61,78,79] or the acquisition of gym classes to increase physical activity [80].

Moreover, some distress and disturbance may occur as a result of socio-cultural pressure. According to Aydin, Eser and Korkmaz [6], negative consumer emotions, such as guilt and shame, may arise as a consequence of fast food consumption among individuals with restrained food consumption.

For example, women may avoid wearing shorts or miniskirts to help hide their legs, while men choose trousers to disguise their waistline, prefer black-coloured clothing to look thinner or avoid wearing long coats because it may accentuate their shortness [81,82,83,84].

Studies from around the world have also suggested that between 5 and 15% of patients who undertake cosmetic procedures meet the diagnostic criteria for body dysmorphic disorder (BDD) or heightened body dissatisfaction, focused on a specific feature or with the entire body [10,85,86]. According to Henderson-King and Henderson-King (2005), people seek cosmetic surgery motivated by intrapsychic concerns (e.g., wanting to feel better about oneself) and social concerns (e.g., wanting to be less self-conscious around others or to look younger for social or business reasons). Acceptance of cosmetic surgery may be more related to fears about becoming unattractive than hopes of becoming more attractive.

Henderson-King and Henderson-King [87] developed the Acceptance of Cosmetic Surgery Scale (ACSS), with 15 items, which is widely used in recent studies [88] in combination with the SATAQ-4 and BAS-2 scales [63]. The first component (Intrapersonal) of the ACSS comprises five items and represents an attitudinal component related to the self-oriented benefits of cosmetic surgery in the form of increased satisfaction with personal appearance. The five items of the second component (Social) assess the social motivations for the decision to have cosmetic surgery. Five additional items loaded on a third component (Consider) are related to straightforward assessments of the likelihood that the respondent would consider having cosmetic surgery or with conditions such as pain or side-effects that could influence such a decision.

Therefore, the conceptual model proposed in this paper (Figure 1) suggests two hypotheses regarding the inclusion of the intention to make cosmetic procedures in the set of consumer behaviour consequences as a result of high socio-cultural pressure and low body appreciation.

**(H4).**  *Socio-cultural Attitudes towards Appearance (SATAQ-4) is positively correlated with consumers’ intention to engage in some corrective acquisition of products and services, in particular the intention to have cosmetic surgeries (ACSS) due to social, intrapersonal and consider attitudinal components.*

**(H5).**  *Body-image appreciation (BAS-2) is negatively correlated with consumers’ intention to engage in some corrective acquisition of products and services, in particular the intention to undertake cosmetic surgery (ACSS) due to social, intrapersonal and consider attitudinal components.*

#### 2.3.3. Compensatory Consumption and Compulsive Buying Disorder

Compensatory consumption refers to “the desire for, acquisition, or use of products to respond to a psychological need or deficit” [89]. The theoretical root of compensatory consumption is based on the notion of possessions as part of the extended self [90,91]. Building upon social comparison theory and the compensatory consumption literature, Zheng, Baskin and Peng [91] proposed that inferiority, experienced in threatening non-material social comparison situations, motivates consumers to restore their sense of superiority in the material domain by engaging in conspicuous consumption. However, this depends on whether the comparison target is in a competitive or cooperative relationship with the self and whether consumers have a clear and well-articulated self-concept.

Compensatory consumption can evolve to a borderline personality disorder called compulsive buying disorder (CBD), defined as “maladaptive and repetitive buying behaviour that results in marked personal, social and occupational impairment as well as financial difficulties and distress” [92].

There is a gap in the literature because there are very few studies relating socio-cultural pressure on physical appearance and positive body image with the proneness to engage in compulsive buying behaviour [93,94]. However, there is some evidence that socio-culture pressure (SATAQ) is a predictor of compulsive exercise [95,96].

The conceptual model proposed in this paper (Figure 1) therefore suggests two hypotheses regarding the inclusion of compulsive buying in the set of consumer behaviour consequences as a result of negative body image:

**(H6).**  *Socio-cultural Attitudes towards Appearance (SATAQ-4) is positively correlated with consumers’ proneness to engage in compulsive buying behaviour (CBFS scale).*

**(H7).**  *Body-image appreciation (BAS-2) is negatively correlated with consumers’ proneness to engage in compulsive buying behaviour (CBFS scale);*

## 3. Methods

In order to validate the conceptual model of Figure 1, which was theoretically developed in Section 2, this paper conducted an online questionnaire that measured the constructs of the independent variables of the model and their outcomes resulting from socio-cultural pressure and perceived body dissatisfaction.

### 3.1. Measures

The questionnaire comprised the following measures (see also Table 1 and Table 2):

(a) the influence of socio-cultural pressures was measured with 22 items of the Portuguese version of the Socio-cultural Attitudes Towards Appearance Scale-4 (SATAQ-4) developed by Barra et al. [4] based on the original version of Schaefer et al. [38] comprising five dimensions: “Internalisation—Thin/low body fat”—items 3, 4, 5, 8, 9; “Internalisation—Muscle/athletic”—items 1, 2, 6, 7, 10; “Family pressure”—items 11, 12, 13, 14; “Peer pressure”—items 15, 16, 17, 18; “Media pressure”—items 19, 20, 21, 22.
(b) the 10 items of the Portuguese version of the Body Appreciation Scale-2 (BAS-2) of Tylka and Wood-Barcalow [12], developed by Lemoine et al. [97] and validated by several studies [98,99];
(c) the intention to change each one of ten parts of the body (using the scale 1—nothing to 4—a lot): face, hair loss, upper limbs, breasts and nipples, hips, genitals, lower members, generalised overweight, localised overweight and overall physical appearance.
(d) the intention to make corrective changes was measured with 15 items of the Acceptance of Cosmetic Surgery Scale (ACSS) developed by Henderson-King and Henderson-King [87];
(e) in order to measure the Consumer Shopping Avoidance Behaviour (CSAB) due to body dissatisfaction, the authors proposed six items on a five-point Likert scale (1—never to 5—very often): (1) to avoid shopping in physical retail outlets, (2) to prefer e-commerce; (3) to avoid high-street shops; (4) to hide some body parts during shopping; (5) to avoid interaction with retail salespersons; (6) to hurry shopping times (reduce time spent out of home);
(f) overall satisfaction with his/her body (on a 10-point Likert scale);
(g) overall perception of physical appearance evaluated by others (on a 10point Likert scale);
(h) the intention to buy a product/service as way to compensate for body dissatisfaction, with a list of eleven products: cosmetics, diet products, surgeries, hair-loss treatments, hair extensions, luxury products, hair-colour change, beauty treatments, travelling, health and wellness treatments and gymnasium classes;
(i) the proneness to engage in compulsive buying was measured with the Portuguese version of the Compulsive Buying Follow-up Scale (CBFS) of Mattos et al. [92], which contains six self-report multiple-choice items assessing different aspects of compulsive buying over the past four weeks.

### 3.2. Sample

An online questionnaire was shared on social media networks and university mailing lists, using a convenience/snow-ball sampling method. A sample of 134 participants answered the questionnaire, 105 females (78.4%) and 29 males (21.6%). The average age of all sample respondents was 26.99 years old (SD = 10.433) ranging from 18 to 71 years old. In total, 30.5% of respondents had a high school degree, whereas 69.5% had an undergraduate or master’s degree. In terms of disposable monthly income, 61.9% had a household income less than EUR2000 and 38.1% had a higher monthly income. The majority of the respondents lived in the north of Portugal, in cities such as Braga (46.3%), Guimarães (11.2%) and Porto (6%).

The average weight of respondents was w = 64.25 Kg (SD = 13.64) and the average height was h = 1.66 m (SD = 8.82). The body mass index (BMI) was calculated using the formula (BMI = w/h2), so the average BMI was 19.21 (SD = 3.57) and it is positively correlated with age (R2 = 0.293, *p* < 0.001). The World Health Organisation defined BMI < 25 as the limit for not being overweight while BMI > 30 is the lower limit for obesity. The majority of respondents (94.8%) had a BMI below 25 and only two were considered obese.

Only three participants claimed to have a physical handicap of more than 70%. Regarding the overall satisfaction with their self-body image, 11.2% of respondents stated that they had a self-perception of their body image “below the average”, while 14.9% classified their body image “above the average” and the remaining 73.9% self-ascribed to the group of “average body image”. Moreover, considering the lower half of the scale, (below or equal to 5 on a 1 to 10 Likert scale), 15.7% of respondents rated a negative overall satisfaction with their body image (M = 6.95; SD = 1.774; N = 134) while a higher percentage of 25.4% think that others evaluate their physical appearance negatively (M = 6.42; SD = 1.76; N = 134). These results are aligned with the lower limits of the ranges provided by Barra et al. [4], Fiske et al. [3], Karazsia, Murnen and Tylka [2] and Striegel-Moore et al. [5]. Mann–Whitney tests did not reveal significant differences in these variables, contradicting the gender differences found by several authors [30,31,33,34].

## 4. Results and Discussion

Table 1 presents the means and standard deviations of the 22 items of the five dimensions of the Portuguese version of the Socio-cultural Attitudes Towards Appearance Scale-4 (SATAQ-4) developed by Barra et al. [4] and the ten items of the BAS-2 scale. The total score of the SATAQ-4 (M = 54.13) was below the average score (66), which means that, in this sample, the socio-cultural pressure is not strong. The BAS-2 total score (M = 36.6) was above the scale midpoint (30), thus revealing a positive body appreciation level.

However, the total score of the Acceptance of Cosmetic Surgery Scale (ACSS) (see Table 2) was above the scale midpoint (45), which means that the respondents had a favourable attitude towards cosmetic surgeries. When invited to identify the body parts that definitely should be changed, respondents ranked them, in descending order: localised fat (10.4%), hips and buttocks (9.7%), breasts and nipples (9%), lower members (8.2%) and generalised obesity (8.2%). On the other hand, in terms of consumers’ shopping avoidance behaviour (see Table 2), the respondents rated a very low score, which in general means that respondents in the future are willing to shop in physical retail outlets and high-street shops, not avoiding human interaction with the salespersons and keeping the shopping rhythm at a normal pace.

Considering that the items of the Compulsive Buying Follow-up Scale (CBFS) of Mattos et al. [92] are measured on a scale of 1 to 5, where the lowest limit corresponds to higher levels of compulsive buying, we may conclude that this sample shows a low level of CBFS as the total score (M = 26.14) was above the midpoint of scale (17).

Mann–Whitney tests (see Table 3) revealed significant gender differences, in that women rated higher values than men in the following dimensions: “internalisation of thin body”, “media pressure”, “consumer shopping avoidance behaviour” (CSAB) and the three sub-dimensions of the ACSS. These differences confirm the previous claims about the prevalence of body dissatisfaction among women made by Frederick, Peplau and Lever [30] and the media-pressure effect due to body talk [34] or advertising [55] but contradicted Huang, Peng and Ahn [54], who found that the gender proportion in the sample does not significantly moderate the effect size of media pressure.

In addition, in the right side, Table 3 also presents the results of Mann–Whitney tests between the two sub-samples with high versus low values of BAS-2, after a median split of the sample (median = 3.70).

Although there was no significant difference in the body mass index, as expected, respondents with low values of body appreciation stated higher scores for all dimensions of socio-culture pressure (SATAQ) except for athletic body internalisation. This sub-sample also rated higher values of social-interaction avoidance (CSAB) and lower values of compulsive buying behaviour.

Moreover, for different levels of buying frequency (measured in times per year), Table 4 presents the percentages of respondents that in the past made purchases as a compensation for body dissatisfaction. Considering the product categories, which were used as self-gift compensation more than seven times per year, 26.1% respondents bought cosmetics, 22.4% had beauty treatments, 13.4% bought a luxury product and 11.2% made a hair change. Chi-square tests revealed that there were significant associations between gender and the acquisition of cosmetics (Chi-square = 13.264; *p* = 0.021), aesthetic surgeries (Chi-square = 6.922; *p* = 0.009), hair changes (Chi-square = 14.511; *p* = 0.006) and beauty treatments (Chi-square = 23.871; *p* < 0.001).

In order to validate the hypotheses of the conceptual model of Figure 1, this study calculated the Spearman correlation coefficients between all the dimensions (see Table 5). Furthermore, the authors calculated a structural equation model with IBM AMOS 27.0.0. using the generalised least squares method, which converged with very good fit indices (CMIN = 63.515; DF = 40; *p* = 0.01; RMR = 0.199; GFI = 0.912; AGFI = 0.862; PGFI = 0.58; RMSEA = 0.063).

**Figure 2:**
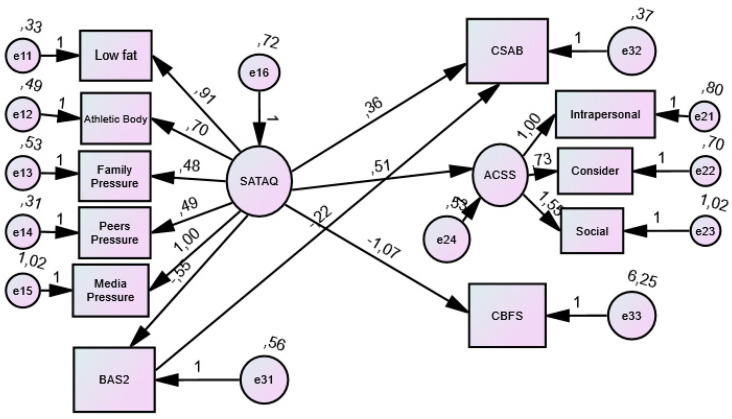
SEM model results: generalized least squares estimates of regression weights.

For SATAQ-4 and ACSS, Table 5 shows that between the latent variable and its dimensions, there are significant Spearman correlation coefficients higher than 0.50. Table 6 presents the factor loadings and errors, while Table 7 and Figure 2 present the generalised least squares estimates of the regression weights.

The average variance extracted (AVE) is the convergent validity test that explains the degree to which items are shared between constructs [100]. To attain this validity, the value of AVE must be greater than or equal to 0.5 [101]. In AMOS the average variance extracted (AVE) is calculated manually using the formula (summation of K2)/n, where K = factor loading (see Table 6) and n = the number of items. Both values of AVE were near the limit (for SATAQ4, AVE = 0.42 and for ACSS, AVE = 0.48).

If the correlation value between the two constructs is less than the square root of the AVE value, discriminant validity exists [102]. For SATAQ4, only for two dimensions (media pressure and internalization of thin/low body fat) were the Spearman correlations (see Table 5) with the latent variable slightly higher than the square root of AVE (0.64).

The internal consistency was measured with Cronbach alpha coefficients, which must be greater than 0.70. Table 1 and Table 2 show that for SATAQ4 and ACSS, Cronbach alphas were all higher than 0.789. A value of composite reliability CR ≥ 0.7 is required to achieve construct reliability [103]. Using the data of Table 6, the calculation of CR obtained values above the required limit (for SATAQ-4, CR = 0.78; for ACSS, CR = 0.74).

The hypothesis H1 was supported by the results, because the socio-cultural pressure influenced body appreciation, with a negative regression weight (−0.546), which means that higher socio-cultural pressures will have a negative impact on the level of body appreciation, as has been claimed in several studies [62,63,64], but contradicting the findings of Lunde [104]. As expected, BMI was positively correlated with family pressure, peer pressure and SATAQ 4 [7], but not with BAS-2, as found by Góngora et al. [105] and Sundgot-Borgen et al. [64].

The impact of socio-cultural pressure on consumers’ avoidance of social/human interaction in a shopping context (CSAB) postulated in hypothesis H2 was supported by the results of the SEM. Respondents exposed to higher socio-cultural pressure rated higher values of the items related to the avoidance to shop in off-line retail outlets and high-street shops. Respondents may want to avoid stigmatisation and shame resulting from the interactions with both retail salespersons and consumers [68,69].

There is also a negative regression weight between body appreciation and CSAB, thus supporting hypothesis H3. As expected, respondents with higher body appreciation levels did not have concerns with the social visibility and interaction with retail salespersons in an off-line retail environment.

The results are also partially aligned with the findings of Jovic et al. [63], Lunde [104] and Meskó and Láng [88], who found a significant positive correlation between the internalisation of thin/low fat body dimension of SATAQ and the intrapersonal dimension of ACSS. Therefore, hypothesis H4 was fully supported by the results, because there were positive correlations between the total score of SATAQ and all the dimensions of ACSS. However, hypothesis H5 was rejected because there was not any influence of body appreciation (BAS-2) on the dimensions of the attitude toward cosmetic surgeries (ACSS), thus contradicting the findings of Meskó and Láng [88].

Hypothesis H6, claiming an influence of socio-cultural pressure on the proneness to shop compulsively, was supported by the negative regression weight estimated in the SEM results. Although there was a positive correlation between CBFS (which has a reversed scale) and BAS-2 (R2 = 0.24, *p* < 0.01), confirming that respondents with positive body image are less prone to engage in compulsive shopping, hypothesis H7 was also rejected by the SEM results, because no significant regression weight between the two constructs was found in the tested models.

## 5. Conclusions

This paper aims to discuss the outcomes of body talk and socio-cultural pressure for positive body image on purchase decision processes, in particular the changes in consumer behaviour preferences and shopping styles, proneness to avoid human/social interaction in a retail context and proneness to change their shopping basket and engage in corrective/compensatory/compulsive shopping behaviour.

In general, the respondents of this sample rated a positive body appreciation, which is aligned with the low levels of BMI and scores of socio-cultural pressures (near the normal scale’s midpoint). In terms of consumer behaviour outcomes, respondents also rated low intentions of social-interaction avoidance and compulsive buying behaviour and a favourable attitude towards cosmetic surgeries. After a median split that divided the sample into two groups (low versus high body appreciation) some significant differences were found that can explain the significant Spearman correlations between the measured constructs.

### 5.1. Implications for Research

There is a gap in the literature concerning the discussion of consumer behaviour outcomes and marketing mix changes as a consequence of socio-cultural pressure stressing the internalisation of a positive body image/appreciation, which remains unexplored by marketing researchers. The relationship between socio-cultural pressure regarding physical appearance with the proneness to engage in compulsive buying behaviour has been suggested by several studies [93,94] but has never been tested using the scales of SATAQ-4 and BAS-2.

The main contribution of this paper is the development and validation of a conceptual model that has postulated several causal relationships between two independent variables, in particular socio-cultural pressure (from the internalisation of thin/athletic body and the social comparison induced by family, peers and media) and body appreciation and the outcomes of social-interaction avoidance, intention to make corrective surgeries and the proneness to engage in compulsive buying behaviour. Only two hypotheses were not supported by the results of the SEM model, since there was found to be no influence of body appreciation on the acceptance of cosmetic surgeries or on the adoption of compulsive buying behaviour.

### 5.2. Managerial Implications

This paper also provides new insights and recommendations for marketing and retailing managers. Even consumers with a positive body image are exposed to socio-cultural pressure from family, peers and media pushing the internalisation of thin/low fat body ideals (especially for women) and an athletic body ideal (especially for men). The literature has confirmed a negative effect of media exposure on body dissatisfaction, which is partly mediated by social-comparison processing [57,58,59]. The use of thin and attractive models (celebrities) as advertising endorsers is also an important source of media pressure that may increase body dissatisfaction levels.

This paper has confirmed that there is a negative correlation between media pressure and body appreciation (see Table 5) which in turn may explain the positive correlations with social-interaction avoidance (CSAB), acceptance of corrective surgery and other borderline behaviour, such as compulsive behaviour. The concept of social-interaction avoidance implies several negative effects for off-line retailers’ sales volume, as consumers reduce the frequency of their purchases, avoid the social visibility of shopping outside the home, in shopping centres or high streets, hiding their body parts that are the source of body dissatisfaction. Moreover, there is a reduction in the average purchase amount, as consumers reduce the time spent inside shops to avoid interaction with retail salespersons and other shoppers. Therefore, brand managers must be aware of these negative effects when they are making decisions about their strategy for advertising copy.

Brands must assume their social responsibility role and mitigate the pressure for internalisation of thin/athletic bodies as a socio-cultural norm. This role is more relevant in the fashion product categories whose products are designed to satisfy the emotional and self-expression needs, which in turn are determined by the perception of the individual’s body image and appreciation. Any distress or disturbance linked to body dissatisfaction will have repercussions on shopping preferences, since consumers will prefer products that hide body-image issues, avoiding other conspicuous products that highlight sources of body dissatisfaction. Moreover, body dissatisfaction may facilitate the development of psychological disorders such as low self-esteem, interpersonal difficulties, depressed mood, social anxiety, low physical activity, substantial morbidity, stress, substance abuse, anorexia nervosa and bulimia nervosa. Obesity is also associated with severe stigmatisation by their surroundings, since obese people are often negatively stereotyped as lazy and lacking in self-control [68,69]. Brand managers must therefore pre-test their advertising campaigns in order to control the implicit/explicit negative stereotyping bias in advertising content. Brands must contribute to educating the public opinion, promoting the inclusion of all people and the acceptance of human body differences and enhancing self-conscious emotions in response to obesity-related negative stereotypes and behaviour [106]. The findings of this paper are aligned with Brown and Tiggemann [55], who stressed that this concern is more relevant among women who are more exposed to media pressure and also more affected by the internalisation of a thin body as an ideal.

On the other hand, there are several product and services categories whose revenues are fuelled by body dissatisfaction, because they somehow provide corrective or preventive actions that aim to mitigate obesity or other issues related to specific body parts, for example, cosmetic, diet and weight-loss treatments, hair-loss treatments, cosmetic surgeries, hairdressers, beauty treatments, healthy food, health and wellness treatments and gym classes, amongst others.

Brand managers should moderate their marketing aggressiveness and inform consumers of the dangers of the physical and psychological addiction to the consumption of such treatments, sometimes with compensatory purposes, because they can evolve into a compulsive buying behaviour. For example, while dissatisfied consumers may pursue happiness by purchasing luxury products or travelling as a compensatory mechanism to balance their psychological needs, consumers who have high levels of positive body appreciation may also engage in conspicuous, exhibitionist or narcissistic consumption activities.

In conclusion, brand and retail managers must acknowledge the need to adapt all components of the marketing mix to cope with this phenomenon, because consumers make changes in their shopping basket and habits, in terms of their preference for less-conspicuous distribution channels and also their willingness to pay for their products and how they process information and advertising.

### 5.3. Limitations of the Study and Further Research Directions

The current research has several limitations that should be acknowledged. First, the results are based on self-reporting measures collected from a convenience non-representative sample in Portugal. Because of this, our findings cannot be generalised to other populations. Although the anonymity of the answers was assured, respondents tend to incur in a response and non-response bias, underrating items related to the self-assumption of psychological disorders such as compulsive buying behaviour. Further research should also compare the response and behaviour of sub-samples with significant differences in BMI and body appreciation scores and may include some qualitative depth analysis of the psychological effects of societal stigmatisation of obese people. Rather than focus on obesity as the main cause of body dissatisfaction, research should extend the analysis to other body dissatisfaction issues such as ageing, hair loss or skin stretch marks/cellulite. It would be relevant to investigate how consumers deal with these problems. Are they too psychological vulnerable? Why are they willing to buy all sorts of “magical” products, regardless of the price?

Besides the differences in retail habits and product preferences highlighted in this research, it is useful to investigate the role of other components of the marketing mix such as pricing policies and sales promotions, omnichannel advertising, store atmosphere, etc. Therefore, this paper is only one part of a broader research project that also investigated other issues related with the implications of socio-cultural pressure and body image/dissatisfaction. For example, this research has also assessed how consumers process and react to different advertising campaigns that promote physical activity in gyms. The authors aim to discuss the role of self-congruency between consumers and advertising endorsers (obese endorsers versus thin endorsers) through the manipulation of (in)coherence between the final goal of physical activity in gyms and the physical appearance of the endorser.

## Figures and Tables

**Table 1:** Means and standard deviations of the items on the SATAQ-4 scale and BAS-2 scale.

SATAQ-4 Scale Items	M	SD
3- I want my body to look very thin.	2.16	1.221
4- I want my body to look like it has little fat.	3.72	1.141
5- I think a lot about looking thin.	2.81	1.363
8- want my body to look very lean.	1.97	1.201
9- I think a lot about having very little body fat.	2.93	1.394
Internalization of Thin/low body fat (Alpha = 0.851)	13.59	5.016
1- It is important for me to look athletic.	3.30	1.176
2- I think a lot about looking muscular	2.51	1.267
6- I spend a lot of time doing things to look more athletic.	2.31	1.265
7- I think a lot about looking athletic.	2.76	1.333
10- I spend a lot of time doing things to look more muscular.	1.99	1.130
Internalization of muscle/athletic body (Alpha = 0.892)	12.87	5.165
11- I feel pressure from family members to look thinner.	1.64	1.146
12- I feel pressure from family members to improve my appearance.	1.96	1.306
13- Family members encourage me to decrease my level of body fat.	2.10	1.303
14- Family members encourage me to get in better shape.	2.66	1.355
Family pressure (Alpha = 0.811)	8.36	4.092
15- My peers encourage me to get thinner.	1.70	1.004
16- I feel pressure from my peers to improve my appearance.	1.61	0.965
17- I feel pressure from my peers to look in better shape.	1.63	1.001
18- I get pressure from my peers to decrease my level of body fat.	1.49	0.882
Peers pressure (Alpha = 0.874)	6.43	3.287
19- I feel pressure from the media to look in better shape.	3.39	1.430
20- I feel pressure from the media to look thinner.	2.93	1.571
21- I feel pressure from the media to improve my appearance.	3.48	1.439
22- I feel pressure from the media to decrease my level of body fat.	3.08	1.542
Media pressure (Alpha = 0.948)	12.88	5.566
Total SATAQ-4- scale score (Alpha = 0.893)	54.13	15.389
Body Appreciation Scale (BAS-2) scale items	M	SD
1. I respect my body.	4.06	0.763
2. I feel good about my body.	3.43	0.896
3. Ifeel that my body has at least some good qualities.	3.88	0.766
4. I take a positive attitude towards my body.	3.64	0.945
5. I am attentive to my body’s needs.	3.69	0.903
6. I feel love for my body.	3.56	0.985
7. I appreciate the different and unique characteristics of my body.	3.48	0.940
8. My behavior reveals my positive attitude toward my body, for example I hold my head high and smile.	3.62	1.046
9. I am comfortable in my body.	3.60	0.926
10. I feel like I am beautiful even if I am different from media images of attractive people (e.g.,models, actresses/actors).	3.63	0.970
Total BAS-2- scale score (Alpha = 0.936)	36.60	7.308

**Table 2:** Means and standard deviations of the items of the ACSS scale, Consumer’s Shopping Avoidance Behaviour (CSAB) and Compulsive Buying Follow-up Scale (CBFS).

Acceptance of Cosmetic Surgery Scale—ACSS Items	M	SD
1- It makes sense to have minor cosmetic surgery rather than spending years feeling bad about the way you look	5.03	1.823
2- Cosmetic surgery is a good thing because it can help people feel better about themselves	5.02	1.596
4- People who are very unhappy with their physical appearance should consider cosmetic surgery as one option	4.23	1.622
5- If cosmetic surgery can make someone happier with the way they look, then they should try it	4.66	1.517
14- Cosmetic surgery can be a big benefit to people’s self-image	4.89	1.733
Intrapersonal (Alpha = 0.885)	23.83	6.876
9- I would seriously consider having cosmetic surgery if my partner thought it was a good idea	1.72	1.323
11- I would think about having cosmetic surgery in order to keep looking young	2.24	1.523
12- If it would benefit my career I would think about having plastic surgery	2.40	1.659
13- I would seriously consider having cosmetic surgery if I thought my partner would find me more attractive	1.65	1.334
15- If a simple cosmetic surgery procedure would make me more attractive to others, I would think about trying it	2.46	1.809
Consider (Alpha = 0.760)	10.46	5.501
3- In the future, I could end up having some kind of cosmetic surgery	3.72	1.885
6- If I could have a surgical procedure done for free I would consider trying cosmetic surgery	3.90	2.184
7- If I knew there would be no negative side effects or pain, I would like to try cosmetic surgery	4.10	2.159
8- I have sometimes thought about having cosmetic surgery	3.00	2.254
10- I would never have any kind of plastic surgery (R)	4.50	1.847
Social (Alpha = 0.907)	19.22	8.851
ACSS total score (Alpha = 0.905)	53.51	17.416
Consumer’s Shopping Avoidance Behavior—CSAB (elaborated by authors)	M	SD
1- intention to avoid shopping in physical retailing outlets	1.51	0.979
2- intention to buy in the e-commerce channel	1.22	0.801
3- intention to avoid to high street shops	1.32	0.881
4- intention to hide some body’s part during shopping	1.94	1.231
5- intention to avoid interaction with shops salespersons	1.34	0.885
6- intention to hurry the shopping (reduce the time out of home)	1.32	0.864
CSAB total score (Alpha = 0.905)	8.66	4.695
Compulsive Buying Follow-up Scale (CBFS)	M	SD
1- In the last 4 weeks, what was the frequency of your compulsive buying episodes?	4.54	0.732
2- In the last 4 weeks, what was the longest period in which you bought compulsively?	4.57	0.630
3- In the last 4 weeks, how much money did you spend buying compulsively	4.67	0.572
4- In the last 4 weeks, how strong was your urge to shop?	3.89	0.846
5- In the last 4 weeks, how have you felt in relation to your debt? (*)	3.76	0.577
6- In the last 4 weeks, how intense was the emotional problem caused by your buying behavior (suffering, anguish, guilt, shame, embarrassment)?	4.71	0.681
CBFS total score (Alpha = 0.789)	26.14	2.847

Legend: (*) Four-point Likert Scale.

**Table 3:** Mann–Whitney tests for differences in scale dimensions’ mean scores between gender sub-samples and low versus high levels of body appreciation (BAS-2).

		N	M	SD	Z (*p*)	BAS	N	M	SD	Z (*p*)
Thin body internalization	Female	105	2.8248	1.00793	−2.380	Low	70	2.9771	1.04925	−2.884
	Male	29	2.3310	0.89964	*p* = 0.017	High	64	2.4344	0.87327	*p* = 0.004
Athletic body internalization	Female	105	2.4933	1.00425	n.s.	Low	70	2.6714	1.07193	n.s.
	Male	29	2.8621	1.10078		High	64	2.4656	0.98565	
Family pressure	Female	105	2.1214	1.04589	n.s.	Low	70	2.3036	1.00532	−2.897
	Male	29	1.9741	0.94336		High	64	1.8555	0.99782	*p* = 0.004
Peer pressure	Female	105	1.5762	0.81388	n.s.	Low	70	1.8821	0.93029	−4.081
	Male	29	1.7241	0.85394		High	64	1.3086	0.55051	*p* < 0.001
Media pressure	Female	105	3.4310	1.32037	−3.182	Low	70	3.6107	1.20809	−3.244
	Male	29	2.4569	1.39685	*p* = 0.001	High	64	2.7930	1.46083	*p* = 0.001
SATAQ-4	Female	105	2.4893	0.69497	n.s.	Low	70	2.6890	0.70707	−4.098
	Male	29	2.2697	0.69891		High	64	2.1714	0.58487	*p* < 0.001
BAS-2	Female	105	3.6124	0.73481	n.s.					
	Male	29	3.8310	0.70158						
CSAB	Female	105	1.5000	0.83141	−2.997	Low	70	1.7071	0.96327	−5.135
	Male	29	1.2414	0.53561	*p* = 0.003	High	64	1.1563	0.33972	*p* < 0.001
ACSS intrapersonal	Female	105	4.9257	1.29325	−2.261	Low	70	4.8229	1.19881	n.s.
	Male	29	4.1862	1.52590	*p* = 0.024	High	64	4.7031	1.55287	
ACSS consider	Female	105	1.9562	0.96436	−2.019	Low	70	2.1771	1.24064	n.s.
	Male	29	2.5862	1.40502	*p* = 0.044	High	64	2.0000	0.92376	
ACSS social	Female	105	4.0362	1.78601	−2.471	Low	70	4.0686	1.81679	n.s.
	Male	29	3.1448	1.54633	*p* = 0.013	High	64	3.5969	1.69761	
ACSS	Female	105	3.6394	1.11158	n.s.	Low	70	3.6895	1.18780	n.s.
	Male	29	3.3057	1.31276		High	64	3.4333	1.12506	
CBFS Total	Female	105	26.2095	2.85783	n.s.	Low	70	25.5857	3.15995	−2.448
	Male	29	25.8966	2.84536		High	64	26.7500	2.33673	*p* = 0.014
BMI	Female	105	18.5048	3.39107	−4.828	Low	70	19.1440	3.74222	n.s.
	Male	29	21.7752	3.04891	*p* < 0.001	High	64	19.2876	3.40933	

**Table 4:** Percentages of respondents that in the past made purchases as a compensation for body image dissatisfaction.

	Never	Rarely (1 to 3 Times Per Year)	Sometimes (4 to 6 Times Per Year)	Less than Once a Month (7 to 11 Times Per Year)	1 to 3 Times Per Month (12 to 36 Times Per Year)	Every Week (52 Times Per Year)	Very often (More than Once a Week)	Total
Cosmetics	26.9	23.1	23.9	12.7	10.4	3.0		100.0
Diet	71.6	21.6	2.2	2.2	1.5		0.7	100.0
Surgeries	97.0	3.0						100.0
Hair-loss treatment	74.6	7.5	10.4	3.7	2.2	0.7	0.7	100.0
Hair extensions	98.5	1.5						100.0
Luxury products	40.3	31.3	14.9	11.2	2.2			100.0
Hair change	41.8	26.1	20.9	9.0	2.2			100.0
Beauty treatment	44.0	25.4	8.2	11.9	9.0	1.5		100.0
Travelling	53.7	22.4	19.4	3.7		0.7		100.0
Wellness	81.3	11.2	5.2	1.5	0.7			100.0
Gymnasium	44.8	26.1	10.4	6.7	1.5	1.5	9.0	100.0

**Table 5:** Spearman correlation coefficients between the model constructs.

	BMI	Thin Ideal	Athletic Ideal	Family Pressure	Peer Pressure	Media Pressure	SATAQ-4	BAS-2	CSAB	ACSS Intrapersonal	ACSS Consider	ACSS Social	ACSS_ Total
BMI	1.000												
Low fat		1.000											
Athletic		0.533 **	1.000										
Family pressure	0.345 **	0.196 *		1.000									
Peers pressure	0.301 **	0.237 **	0.221 *	0.458 **	1.000								
Media pressure		0.436 **	0.218 *	0.278 **	0.271 **	1.000							
SATAQ-4	0.176 *	0.739 **	0.579 **	0.535 **	0.577 **	0.745 **	1.000						
BAS-2		−0.286 **		−0.263 **	−0.336 **	−0.295 **	−0.375 **	1.000					
CSAB		0.366 **	0.191 *	0.324 **	0.335 **	0.422 **	0.493 **	−0.531 **	1.000				
ACSS Intrapersonal		0.216 *	0.175 *			0.300 **	0.234 **			1.000			
ACSS Consider		0.267 **	0.202 *	0.241 **		0.171 *	0.317 **			0.436 **	1.000		
ACSS Social		0.302 **	0.209 *			0.288 **	0.325 **		0.261 **	0.581 **	0.496 **	1.000	
ACSS Total		0.309 **	0.232 **			0.322 **	0.358 **		0.238 **	0.813 **	0.715 **	0.891 **	1.000
CBFS		−0.245 **	−0.260 **		−0.175 *	−0.242 **	−0.322 **	0.240 **	−0.172 *		−0.255 **	−0.276 **	−0.276 **

Legend: * *p* < 0.05; ** *p* < 0.01.

**Table 6:** Factor loadings and errors of constructs.

			Loadings	Errors
ACSS	<---	SATAQ	0.514	0.725
BAS2	<---	SATAQ	−0.527	0.53
Media_Pressure	<---	SATAQ	0.644	0.562
Peers_Pressure	<---	SATAQ	0.605	1.024
Family_Pressure	<---	SATAQ	0.488	0.308
Muscle_Athletic	<---	SATAQ	0.646	0.491
Thin_low_fat	<---	SATAQ	0.803	0.331
ACSS_intrapersonal	<---	ACSS	0.689	0.797
ACSS_consider	<---	ACSS	0.593	0.704
ACSS_social	<---	ACSS	0.793	1.022
CSAB	<---	SATAQ	0.405	0.374
CBFS_total	<---	SATAQ	−0.341	6.252
CSAB	<---	BAS2	−0.262	0.525

**Table 7:** SEM generalized least squares estimates of regression weights.

			Estimate	S.E.	C.R.	*p*	Hypothesis
ACSS	<---	SATAQ	0.512	0.133	3.865	***	H4- supported
BAS2	<---	SATAQ	−0.546	0.108	−5.035	***	H1- supported
Media_Pressure	<---	SATAQ	1.000				
Peers_Pressure	<---	SATAQ	0.495	0.101	4.902	***	
Family_Pressure	<---	SATAQ	0.476	0.118	4.041	***	
Muscle_Athletic	<---	SATAQ	0.697	0.143	4.880	***	
Thin_low_fat	<---	SATAQ	0.910	0.143	6.346	***	
ACSS_intrapersonal	<---	ACSS	1.000				
ACSS_consider	<---	ACSS	0.728	0.145	5.036	***	
ACSS_social	<---	ACSS	1.548	0.271	5.713	***	
CSAB	<---	SATAQ	0.360	0.097	3.710	***	H2- supported
CBFS_total	<---	SATAQ	−1.065	0.325	−3.276	0.001	H6- supported
CSAB	<---	BAS2	−0.225	0.079	−2.861	0.004	H3- supported

Legend: *** *p* < 0.001.

## Data Availability

Not applicable.
